# The Protective Effect of Low-Dose Aspirin against Colorectal Cancer Is Unlikely Explained by Selection Bias: Results from Three Different Study Designs in Clinical Practice

**DOI:** 10.1371/journal.pone.0159179

**Published:** 2016-07-18

**Authors:** Lucía Cea Soriano, Montse Soriano-Gabarró, Luis A. García Rodríguez

**Affiliations:** 1 Spanish Centre for Pharmacoepidemiologic Research, Madrid, Spain; 2 Epidemiology, Bayer Pharma AG, Berlin, Germany; Baylor University Medical Center, UNITED STATES

## Abstract

**Background:**

We conducted three differently designed nested case–control studies to evaluate whether the protective effect of low-dose aspirin against colorectal cancer (CRC) is explained by selection bias.

**Methods:**

Using a large validated UK primary care database, we followed different cohorts of patients, who varied in their demographic and clinical characteristics, to identify first ever cases of CRC. In Studies 1 and 2, two cohorts were followed, i) new users of low-dose aspirin at start of follow-up (N = 170,336 in Study 1, N = 171,527 in Study 2) and either ii) non-users of low-dose aspirin (Study 1, N = 170,336) or new users of paracetamol (Study 2, N = 149,597) at start of follow-up. In Study 3 a single cohort of individuals näive to low-dose aspirin at the start of observation was followed. Controls were selected using incidence sampling and logistic regression used to obtain an unbiased estimate of the incidence rate ratio (RR) with 95% confidence intervals (CIs). Low-dose aspirin exposure was analyzed ‘as-treated’ before the index date (CRC date for cases, random date for controls).

**Results:**

In the three studies, median (maximum) follow-up was 5.1 (12), 5.8 (12) and 7.5 (13) years, respectively. 3033 incident CRC cases were identified in Study 1, 3174 in Study 2, and 12,333 in Study 3. Current use of low-dose aspirin was associated with a significantly reduced risk of 34%, 29% and 31% in the three studies, respectively; corresponding RRs (95% CIs) were 0.66 (0.60–0.73), 0.71 (0.63–0.80) and 0.69 (0.64–0.74). In each study, significantly reduced risks of CRC were seen when low-dose aspirin was used for primary or secondary cardiovascular disease prevention, in both sexes, and across all age groups evaluated.

**Conclusion:**

Low-dose aspirin is associated with a significantly reduced risk of CRC. The consistency of our findings across different studies makes selection bias an unlikely explanation.

## Introduction

Colorectal cancer (CRC) is an increasingly global public health problem and is the third most commonly diagnosed cancer worldwide [1]. Prognosis is highly related to stage at diagnosis [2]; in the UK, 5-year relative survival is over 90% for patients with Dukes Stage A CRC at diagnosis compared with around 50% and less than 7% for patients presenting with Stage C and D CRC, respectively [3]. Comparable estimates have been reported in the US [4].

Evidence from cardiovascular trials suggests daily use of low-dose aspirin reduces the risk of developing CRC, and that part of this reduction in risk might be due to protection against metastatic disease [5,6]. Evidence from patients in the ‘real-world’ involve pharmacoepidemiological studies, which require a robust and unbiased study design to obtain valid results. In order to rigorously investigate the relationship between low-dose aspirin and risk of CRC, we carried out three separate cohort studies with nested case–control analysis, each adopting a different study design. We hypothesized that low-dose aspirin would be associated with a reduction in risk in all three studies, thereby minimizing the chance that the protective effect is due to selection bias. The study protocol was reviewed and approved by an independent scientific review committee (reference number 12-044V).

## Materials and Methods

### Data source

**Fig 1:**
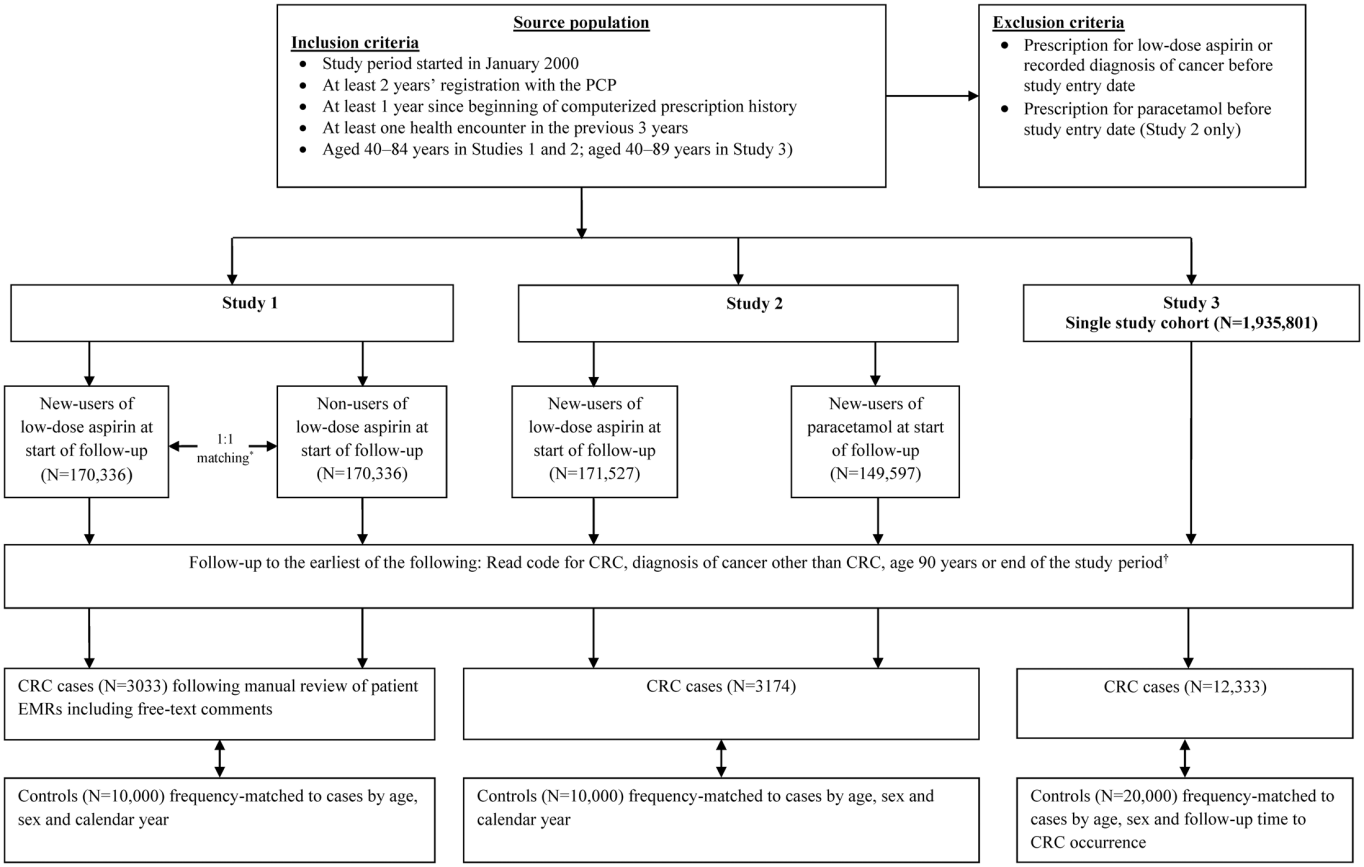
Identification of the study cohort(s) in each study design. *Matched by age, sex and number of PCP visits. ^†^31 December 2011 in Study 1 and 31 December 2012 in Studies 2 and 3. CRC, colorectal cancer; PCP, primary care pracitioner.

We used data from The Health Improvement Network (THIN), a validated primary care database of anonymized patient records in the United Kingdom (UK). Almost all of the UK population are registered with a primary care practitioner (PCP) and THIN is representative of the UK population with regards to age, sex and geographic distribution [7, 8]. Primary care practitioners record information electronically as part of routine patient care, and send it to THIN for use in research projects. Currently close to 600 general practices throughout the UK have contributed data to THIN [9]. Details of the database, which has been used for a large number of observational studies, have been described previously [10]. THIN was used for each study although the study periods varied slightly owing to the data available at the time each study was conducted (Fig 1). For each study, individuals entered the source population upon meeting all eligibility criteria (S1 Text).

### Source population and identification of study cohort(s) in each study design

The source population comprised individuals in THIN meeting data quality and completion standards (Fig 1). Individuals were excluded if they had a prescription for low-dose aspirin (75 or 300 mg; tablets available in the UK) or a recorded diagnosis of cancer any time before study entry. For Study 2, individuals were also excluded if they had a prescription for paracetamol monotherapy prior to study entry; this was not an exclusion criterion in Studies 1 and 3. Identification of the study cohort(s) in the three different studies is illustrated in Fig 1 with further details can be found in the S1 Text. In the first study (Study 1) a cohort of new-users of low-dose aspirin was identified with the date of each individual’s first low-dose aspirin prescription set as the start of follow-up (start date). Each member of this cohort was matched to an individual who was still free of low-dose aspirin at the start date. Matching was by age, sex and number of PCP visits in the previous year. The second study (Study 2) was similar in design to Study 1, but here the second cohort comprised new users of a ‘neutral’ drug—paracetamol—at the start date in order to minimize any potential healthy user bias arising from differences between users and non-users of low-dose aspirin. Unlike in Study 1, in this study, individuals in the new users of low-dose aspirin cohort were not matched to the new users of paracetamol cohort. In this study, paracetamol (used as monotherapy) was chosen as a suitable comparison drug to low-dose aspirin by virtue of meeting the following necessary criteria: i) it has no known effect, either positive or negative, on the risk of CRC, ii) it is not a proxy of significant co-morbidity, and iii) prevalence of use among the source population is sufficiently common to sample from. In the third study (Study 3), we used a more conventional methodology using a cohort of all individuals in the source population rather than the selection of two separate cohorts.

### Follow-up to identify incident cases of CRC

In each study, members of the study cohort(s) were followed until the earliest of the following endpoints: occurrence of a Read code for CRC (S1 Table; identified through computerized searches), a recorded diagnosis of cancer other than CRC, age of 90 years, death or the end of the study period (31 December 2011 in Study 1 and 31 December 2012 in Studies 2 and 3; Fig 1). For patients with a diagnostic Read code for CRC in Study 1, 86.4% were deemed to be true incident cases following manual review of the patients’ electronic medical records (EMRs) [11]. The index date was the date of first CRC-related symptom, screening or diagnostic procedure, or surgery, whichever came first. Because of the high positive predictive value of the CRC Read codes in THIN in Study 1, we did not manually review the EMRs of patients with a Read code for CRC in Studies 2 and 3, and the index date in these two studies was the date of the computer-detected CRC Read code.

### Selection of controls

In each study, controls were selected to perform nested case–control analyses. In Studies 1 and 2, 10,000 controls were randomly sampled and frequency matched to CRC cases by age, sex and calendar year. In Study 3, a total of 20,000 controls were randomly sampled and frequency-matched to CRC cases by age, sex and follow-up time to CRC occurrence. We used incidence density sampling so that the likelihood of being selected as a control was proportional to the person-time at risk. To do this, we generated a random date within the study period for each member of the study cohort(s). If this date was included in the patients’ eligible person-time, we used their random date as the index date and marked that person as an eligible control. Controls were subject to the same eligibility criteria applied to cases.

### Identification of risk factors

Information on potential risk factors was extracted from the database, including demographics, body mass index (BMI), lifestyle factors, morbidities, medication use, and PCP visits, referrals and hospitalizations. Medication use was classified into three categories: *current use*, when supply of the most recent prescription lasted until the index date or ended 1–90 days before the index date; *recent/past use*, when supply of the most recent prescription ended ≥91 days before the index date; and *non-use*, when there was no recorded use at any time before the index date. Low-dose aspirin was analyzed using an ‘as-treated’ approach, evaluating exposure between the start and index dates, and therefore for Studies 1 and 2 was irrespective from the study cohort from which the individual was assigned at start of follow-up (further details can be found in the S1 Text). The reason we did not use an intention-to-treat analysis in Studies 1 and 2 (i.e. by low-dose aspirin exposure at start of follow-up) comparing incidence rates of CRC between the two study cohorts, was because use of low-dose aspirin could have changed in a substantial number of patients over the lengthy follow-up duration; intention-to-treat analysis was not applicable in Study 3. All medication use evaluated (including clopidogrel and dual antiplatelet therapy [DAT] with clopidogrel/low-dose aspirin) was also analyzed ‘as-treated’, according to the three exposure categories above. Further methods on the evaluation of medications (including low-dose aspirin) and other risk factors can be found in the S1 Text.

### Statistical analysis

In each study, incidence rates of CRC were calculated using the number of cases as the numerator and the respective person-time as the denominator, stratified by sex and age group. Nested case–control analyses were performed to estimate the association between low-dose aspirin and the occurrence of CRC. Under the study design of incidence density sampling, the odds ratio is an unbiased estimator of the incidence rate ratio (RR) [12]. Rate ratios and 95% confidence intervals (CIs) were calculated by unconditional multiple logistic regression models adjusted in each study for the frequency matching factors, number of PCP visits, smoking, non-steroidal anti-inflammatory drugs and BMI. Use of insulin and oral steroids were also included in the fully adjusted model in Studies 1 and 2, with adjustment for paracetamol monotherapy also undertaken in Study 2. All potential confounders were treated as categorical variables and a separate level was created for variables with missing information. Stratified analyses and tests for interaction were performed (S1 Text). Statistical analyses were carried out using STATA package version 12.0 (StataCorp LP, College Station, TX, USA).

## Results

### Characteristics of the study cohort(s)

Characteristics of the study cohort(s) in each of the three studies are shown in Table 1. As expected, due to the more restrictive inclusion criteria in Studies 1 and 2, the mean age at start of follow up was higher (64 years) in these two studies than in Study 3, while a greater proportion of younger individuals were present in Study 3 (mean age 53 years). For the same reason, follow-up to identify CRC generally started from an earlier calendar period in Study 3. The distribution of men was slightly higher in Study 1 (52%), than in Study 2 (48%) and Study 3 (49%). Patient characteristics of the study cohort(s) in each study are shown in Table 2. As expected, individuals in the study cohort of Study 3 had fewer comorbidities, were less likely to be overweight, obese, taking the medications evaluated, and had fewer PCP visits than individuals in Studies 1 and 2. In Study 1, the low-dose aspirin cohort were less healthy in terms of comorbidities, BMI, smoking status and previous colonoscopy/sigmoidoscopy procedures than the non-user of low-dose aspirin at start of follow-up cohort, whereas in Study 2, the new users of low-dose paracetamol cohort had a higher level of several comorbidities (chronic obstructive pulmonary disease, inflammatory bowel disease, depression, gastrointestinal conditions), more previous colonoscopy/sigmoidoscopy procedures and a similar level of current smokers compared with the low-dose aspirin cohort. In addition, the prevalence of these particular patient characteristics in the new users of paracetamol at start of follow-up cohort in Study 2 was higher than those in the non users of low-dose aspirin at start of follow-up cohort in Study 1.

**Table 1:** Characteristics of study cohort(s) in each study design.

	Study design		
	Study 1	Study 2	Study 3
	Two cohorts: new-users of low-dose aspirin at start of follow-up (N = 170,225) and non-users (N = 169,992) at start of follow-up *	Two cohorts: new-users of low-dose aspirin at start of follow-up (N = 171,510) and new users of paracetamol (N = 149,576) at start of follow-up*	Single cohort, (N = 1,935,801)
**Calendar year at start of follow-up, n (%)**			
2000–2003	114,487 (33.7)	103,100 (32.1)	1,013,179 (52.3)
2004–2006	114,958 (33.8)	109,167 (34.0)	362,963 (18.8)
2007–2009/2012^†^	110,772 (32.6)	108,819 (33.9)	559,653 (28.9)
**Men, n (%)**	178,274 (52.4)	153,676 (47.9)	897,615 (46.4)
**Age at start of follow-up (years)**			
Mean	63.9	63.4	52.5
Median	64.0	64.0	49.0
**Follow-up duration (years)**^†^			
Median	5.1	5.8	7.5
Mean (sd; range)^†^	5.3 (2.9; 0.003–11.99)	6.0 (3.1; 0.003–12.99)	7.3 (4.17; 0.003–12.99)
**Number for incident CRC cases**	3033	3174	12,333

CRC, colorectal cancer.

*For Studies 1 and 2, the total numbers at start of follow-up in each cohort differ from those at the end of study cohort enrollment as shown in Fig 1 (N = 170,336 in both cohorts in Study 1, and N = 171,527 in the new-user low-dose aspirin at start of follow-up cohort and N = 149,576 in the new-user paracetamol at start of follow-up cohort in Study 2). This is because, in these two studies, an updated version of THIN (the latest data released) was used at the start of follow-up to identify CRC cases, whereas an earlier version was used for the ascertainment of study cohorts. For these two studies, some patients included in the study cohort(s) were no longer eligible for inclusion in the follow-up to identify CRC cases for reasons such as they were no longer included in the database (e.g. had transferred out of the practice); it transpired they had died on the same day that they became an eligible study cohort member; they no longer met all inclusion criteria (e.g. data incompleteness).

^†^Follow-up was up to 31 December 2012 in Studies 2 and 3, and up to 31 December 2011 in Study 1.

**Table 2:** Demographics, comorbidities, lifestyle characteristics and healthcare utilization of individuals in the study cohort(s) in each study.

	Study design				
Characteristic	Study 1		Study 2		Study 3
	Non-users of low-dose aspirin at start of follow-up cohort	New users of low-dose aspirin at start of follow-up cohort	New-users of paracetamol at start of follow-up cohort	New-users of low-dose aspirin at start of follow-up cohort	Single cohort
	N = 169,992	N = 170,225*	N = 149,576	N = 171,510*	(N = 1,935,801)
	n (%)	n (%)	n (%)	n (%)	n (%)
**Sex**					
Male	89,079 (52.4)	89,079 (52.4)	55,604 (37.1)	98,072 (57.2)	897,616 (46.4)
Female	80,913 (47.6)	80,913 (47.6)	93,972 (62.8)	73,438 (42.8)	1,038,185 (53.6)
**Age group (years)**^†^					
40–59	57,535 (33.8)	57,528 (33.8)	53,106 (35.5)	56,743 (33.1)	1,405,655 (72.6)
60–69	57,499 (33.8)	57,497 (33.8)	47,789 (32.0)	62,127 (36.2)	293,474 (15.2)
70–79	41,927 (24.7)	42,130 (24.7)	36,849 (24.6)	42,101 (24.6)	169,276 (8.7)
80–89	13,031 (7.7)	13,070 (7.7)	11,832 (7.9)	10,539 (6.1)	67,396 (3.5)
**PCP visits**^‡^					
0–1	5842 (3.4)	5887 (3.4)	8,693 (5.8)	6,991 (4.1)	507,903 (26.2)
2–4	21,711 (12.8)	21,770 (12.8)	24,017 (16.1)	25,053 (14.6)	582,691 (30.1)
5–9	49,238 (29.0)	49,312 (29.0)	43,651 (29.2)	53,490 (31.2)	477,061 (24.6)
10–19	65,353 (38.4)	65,404 (38.4)	50,268 (33.6)	64,036 (37.3)	285,849 (14.8)
≥20	27,848 (16.4)	27,852 (16.4)	22,947 (15.3)	21,940 (12.8)	82,297 (4.3)
**Comorbidities**					
Hypertension	55,228 (32.5)	25,041 (50.0)	47,787 (31.9)	85,929 (50.1)	293,880 (15.2)
Diabetes	12,365 (7.3)	34,629 (20.3)	10,314 (6.9)	34,028 (19.8)	66,019 (3.4)
COPD	7891 (4.6)	8786 (5.2)	8,285 (5.5)	7,577 (4.4)	34,509 (1.8)
IBD	2061 (1.2)	2012 (1.2)	2368 (1.6)	1845 (1.1)	18,439 (0.95)
Depression	31,654 (18.6)	37,587 (22.1)	40,700 (27.2)	33,014 (19.2)	359,626 (18.6)
GI conditions	33,161 (19.5)	38,800 (22.8)	37,719 (25.2)	34,391 (20.1)	252,427 (13.0)
**Colonoscopy/sigmoidoscopy**^§^	14,262 (8.4)	15,888 (9.3)	15,366 (10.3)	14,406 (8.4)	92,911 (4.8)
**Smoking**					
Non-smoker	79,726 (46.9)	70,997 (41.7)	67,843 (45.4)	71,195 (41.5)	928,855 (48.0)
Current smoker	31,157 (18.3)	36,441 (21.4)	31,627 (21.1)	36,175 (21.1)	424,713 (21.9)
Former smoker	48,552 (28.6)	54,984 (32.3)	42,099 (28.1)	55,375 (32.3)	337,603 (17.4)
Unknown	10,557 (6.2)	7803 (4.6)	8007 (5.4)	8765 (5.1)	244,630 (12.6)
**BMI (kg/m**^**2**^**)**					
15–19	5976 (3.5)	4605 (2.7)	6,010 (4.0)	4,227 (2.5)	76,336 (3.9)
20–24	47,714 (28.1)	38,612 (22.7)	38,419 (25.7)	39,293 (22.9)	565,770 (29.2)
25–29	57,113 (33.6)	60,180 (35.4)	48,608 (32.5)	62,011 (36.2)	548,393 (28.3)
≥30	31,460 (18.5)	46,875 (27.5)	35,542 (23.8)	44,703 (26.1)	306,849 (15.9)
Unknown	27,729 (16.3)	19,953 (11.7)	20,997 (14.0)	21,276 (12.4)	438,453 (22.6)

All variables were measured anytime before the start of follow-up.

*75.5% (N = 129,464) of the low-dose aspirin cohort in Study 2 were also members of the low-dose aspirin cohort in Study 1.

^†^Age was calculated at start date.

^‡^Number of PCP visits in the year before start of follow-up.

^§^Prior record of colonoscopy, sigmoidoscopy or GI adenoma.

BMI, body mass index; GI, gastrointestinal; IBD, inflammatory bowel disease; PCP, primary care practitioner.

### Incidence of CRC

**Fig 2:**
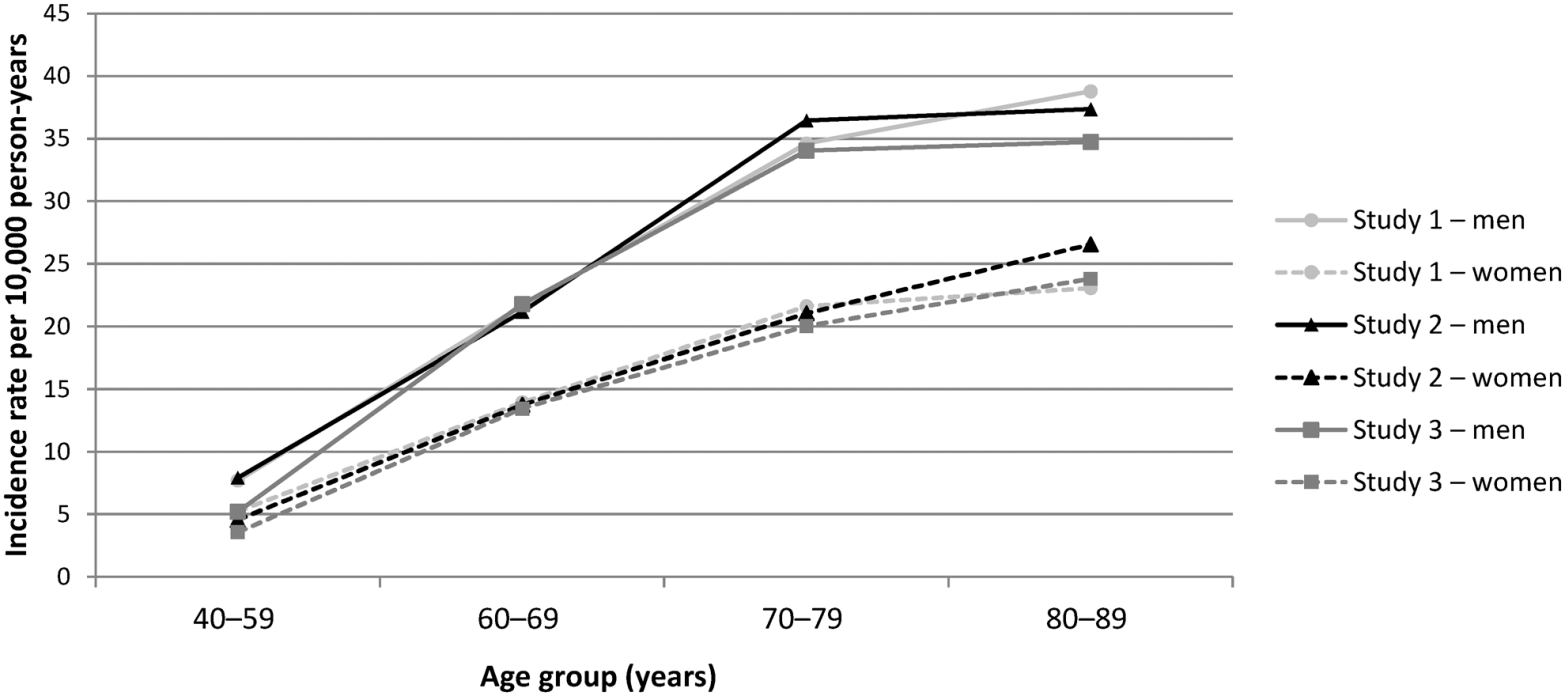
Incidence of CRC by age group and sex for each study design. CRC, colorectal cancer.

A total of 3033 incident CRC cases were identified in Study 1 compared with 3174 in Study 2 and 12,333 in Study 3. Of the 3033 cases in Study 1, 55.2% (n = 1674) were also cases in Study 2 (because they met the eligibility criteria for both studies), while of the 12,333 cases in Study 3, 65.2% (n = 8043) were not cases in Studies 1 or 2. Details on the clinical features of the 3033 cases in Study 1 have been described previously [13]. Median follow up duration was 5.1 years in Study 1, 5.8 years in Study 2 and 7.5 years in Study 3. The incidence of CRC per 10,000 person-years was 16.74 (95% CI: 16.16–17.35), 16.34 (95% CI: 15.78–16.93) and 8.68 (95% CI: 8.53–8.83) in Studies 1, 2 and 3, respectively. In each study, incidence rates increased with age in both sexes and were higher in men than women in all age groups (Fig 2).

### Characteristics of cases and controls

Cases and controls were generally comparable with regards to demographics, healthcare utilization, lifestyle characteristics and comorbidities across the three study designs (Table 3); however, cases had more healthcare visits (PCP visits, referrals and hospitalizations) and higher alcohol consumption. In both Studies 1 and 2, 41% of cases and 45% of controls were current users of low-dose aspirin, compared with 12% cases and 13% controls in Study 3. More than 90% of low-dose aspirin use among cases and controls in each study was at a dose of 75 mg/day (see S2 Table for further details on aspirin use among cases and controls in each study). The proportion of low-dose aspirin current users (among both cases and controls combined) who had at least 1 years’ use of the drug by the index date was 68.0%, 77.3% and 73.6% in Studies 1, 2 and 3, respectively.

**Table 3:** Characteristics of cases and controls in each study design.

	Study design					
Characteristic	Study 1		Study 2		Study 3	
	Cases	Controls	Cases	Controls	Cases	Controls
	N = 3033	N = 10,000	N = 3174	N = 10,000	N = 12,333	N = 20,000
	n (%)	n (%)	n (%)	n (%)	n (%)	n (%)
**Sex**						
Male	1806 (59.5)	5930 (59.3)	1810 (57.0)	5695 (57.0)	6720 (54.5)	10,935 (54.7)
Female	1227 (40.5)	4070 (40.7)	1364 (43.0)	4305 (43.0)	5613 (45.5)	9065 (45.3)
**Age group (years)**						
40–49	19 (0.6)	65 (0.7)	31 (1.0)	114 (1.1)	599 (4.9)	963 (4.8)
50–59	240 (7.9)	779 (7.8)	203 (6.4)	639 (6.4)	1829 (14.8)	2944 (14.7)
60–69	875 (28.8)	2894 (28.9)	866 (27.3)	2737 (27.4)	3682 (29.9)	5980 (29.9)
70–79	1273 (42.0)	4196 (42.0)	1335 (42.1)	4184 (41.8)	4039 (32.7)	6565 (32.8)
80–89	626 (20.6)	2066 (20.7)	739 (23.3)	2326 (23.3)	2184 (17.7)	3548 (17.7)
**Calendar year**						
2000–2003	428 (14.1)	1327 (13.3)	250 (7.9)	815 (8.2)	2286 (18.5)	4008 (20.0)
2004–2006	818 (27.0)	2691 (26.9)	693 (21.8)	2196 (22.0)	2855 (23.1)	4588 (22.9)
2007–2011	1787 (58.9)	5982 (59.8)	2231 (70.3)	6989 (69.9)	7192 (58.3)	11,404 (57.0)
**BMI (kg/m**^**2**^**)**						
<20	111 (3.7)	343 (3.4)	124 (3.9)	331 (3.3)	584 (4.7)	717 (3.6)
20–24	774 (25.5)	2665 (26.7)	797 (25.1	2558 (25.6)	3485 (28.3)	5766 (28.8)
25–29	1144 (37.7)	3793 (37.9)	1209 (38.1)	3790 (37.9)	4269 (34.6)	6934 (34.7)
≥30	714 (23.5)	2324 (23.2)	809 (25.5)	2589 (25.9)	2469 (20.0)	3932 (19.7)
Unknown	290 (9.6)	875 (8.8)	235 (7.4)	732 (7.3)	1526 (12.4)	2651 (13.3)
**Smoking**						
Non-smoker	1231 (40.6)	4378 (43.8)	1234 (38.9)	4302 (43.0)	5557 (45.1)	9592 (48.0)
Current	376 (12.4)	1292 (12.9)	381 (12.0)	1212 (12.1)	1748 (14.2)	2946 (14.7)
Former	1362 (44.9)	4102 (41.0)	1515 (47.7)	4325 (43.2)	4560 (37.0)	6562 (32.8)
Unknown	64 (2.1)	228 (2.3)	44 (1.4)	161 (1.6)	468 (3.8)	900 (4.5)
**Alcohol consumption (units/week)**						
None	483 (15.9)	1771 (17.7)	530 (16.7)	1925 (8.6)	1801 (14.6)	3233 (16.2)
1–9	1401 (46.2)	4722 (47.2)	1498 (47.2)	4746 (47.5)	5524 (44.8)	9040 (45.2)
10–20	480 (15.8)	1521 (15.2)	497 (15.7)	1454 (14.5)	1972 (16.0)	3076 (15.4)
21–41	216 (7.1)	564 (5.6)	209 (6.6)	522 (5.2)	758 (6.2)	1084 (5.4)
≥42	58 (1.9)	161 (1.6)	87 (2.7)	194 (1.9)	334 (2.7)	319 (1.6)
Unknown	395 (13.0)	1261 (12.6)	353 (11.1)	1159 (11.6)	1944 (15.8)	3248 (16.2)
Comorbidities*						
Diabetes	598 (19.7)	1852 (18.5)	686 (21.6)	1873 (18.7)	1424 (11.5)	1915 (9.6)
IBD	40 (1.3)	124 (1.2)	45 (1.4)	132 (1.3)	209 (1.7)	285 (1.4)
IBS	176 (5.8)	534 (5.3)	208 (6.6)	621 (6.2)	787 (6.4)	1100 (5.5)
Gout	250 (8.2)	765 (7.6)	269 (8.5)	836 (8.4)	778 (6.3)	1112 (5.6)
Hypertension	1740 (57.4)	5626 (56.3)	1908 (60.1)	5816 (58.2)	5227 (42.4)	7926 (39.6)
Hypercholesterolemia	484 (16.0)	1655 (16.6)	523 (16.5)	1771 (17.7)	1407 (11.4)	2266 (11.3)
Upper GI disorders^†^	656 (21.6)	2165 (21.6)	730 (23.0)	2293 (22.9)	1888 (15.3)	3074 (15.4)
**PCP visits**^‡^						
0–1	92 (3.0)	486 (4.9)	10 (0.3)	170 (1.7)	237 (1.9)	2138 (10.7)
2–4	261 (8.6)	1094 (10.9)	68 (2.1)	772 (7.7)	1091 (8.8)	3820 (19.1)
5–9	699 (23.0)	2624 (26.2)	427 (13.5)	2,456 (24.6)	2913 (23.6)	5529 (27.6)
10–19	1164 (38.4)	3715 (37.1)	1313 (41.4)	4157 (41.6)	4879 (39.6)	5818 (29.1)
≥20	817 (26.9)	2081 (20.8)	1356 (42.7)	2445 (24.5)	3213 (26.1)	2695 (13.5)
**Referrals**^‡^						
0–1	1182 (39.0)	4760 (47.6)	510 (16.1)	4140 (41.4)	3228 (26.2)	11,829 (59.1)
2–4	917 (30.2)	2778 (27.8)	973 (30.7)	3015 (30.1)	4205 (34.1)	4806 (24.0)
5–9	612 (20.2)	1716 (17.2)	1025 (32.3)	1976 (19.8)	3316 (26.9)	2450 (12.2)
≥10	322 (10.6)	746 (7.5)	666 (21.0)	869 (8.7)	1584 (12.8)	915 (4.6)
**Hospitalizations**^‡^						
None	2424 (79.9)	8496 (85.0)	2000 (63.0)	8303 (83.0)	8629 (70.0)	17,885 (89.4)
1	375 (12.4)	930 (9.3)	650 (20.5)	1005 (10.1)	2250 (18.2)	1389 (6.9)
2	148 (4.9)	328 (3.3)	273 (8.6)	400 (4.0)	832 (6.7)	456 (2.3)
≥3	86 (2.8)	246 (2.5)	251 (7.9)	292 (2.9)	622 (5.0)	270 (1.4)
**Medications**^§^						
Clopidogrel	110 (3.6)	291 (2.9)	123 (3.9)	337 (3.4)	232 (1.9)	312 (1.6)
DAT	54 (1.8)	134 (1.3)	52 (1.6)	152 (1.5)	73 (0.6)	105 (0.5)
Paracetamol monotherapy	393 (13.0)	1209 (12.1)	780 (24.6)	2041 (20.4)	1447 (11.7)	1881 (9.4)

*Any time before the index date.

^†^Any time before the start date.

^‡^In the year before the index date. In the paracetamol comparator study design (Study 2), the number of PCP visits were categorized as 0–5 (replaces category 0–1), 2–4, 6–9 (replaces category 5–9), 10–19 and ≥20.

^**§**^Current use.

BMI, body mass index; DAT, dual antiplatelet therapy; GI, gastrointestinal; IBD, inflammatory bowel disease; IBS, irritable bowel syndrome; PPI, proton pump inhibitor.

### Low-dose aspirin and risk of CRC

**Fig 3:**
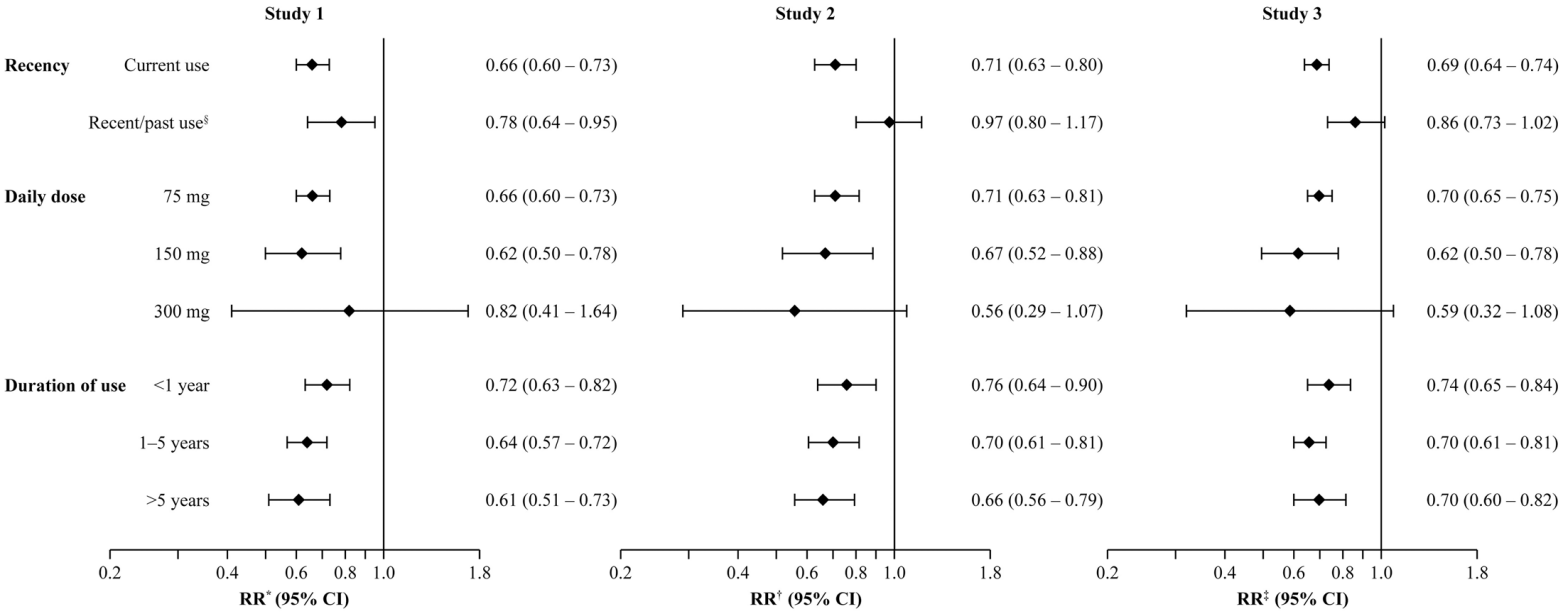
RRs (95% CIs) for risk of CRC associated with use of low-dose aspirin for each study design. All estimates are among current users of low-dose aspirin (versus never users) unless otherwise specified. In the adjusted models, smoking, BMI, use of insulins, NSAIDs, oral-steroids, paracetamol and low-dose aspirin were ascertained any time before the index date. *Adjusted by matching factors, number of PCP visits in the year before the index date, smoking, insulin, NSAIDs, BMI and oral steroids. ^†^Adjusted by matching factors, number of PCP visits in the year before the index date, smoking, BMI, NSAIDs, and paracetamol. ^‡^Adjusted by matching factors, number of PCP visits in the year before the index date, smoking, NSAIDs and BMI. ^§^For patients with a duration of use of at least 1 year. BMI, body mass index; CI, confidence interval; CRC, colorectal cancer; NSAIDs, non-steroidal anti-inflammatory drugs; PCP, primary care practitioner; RR, rate ratio.

**Fig 4:**
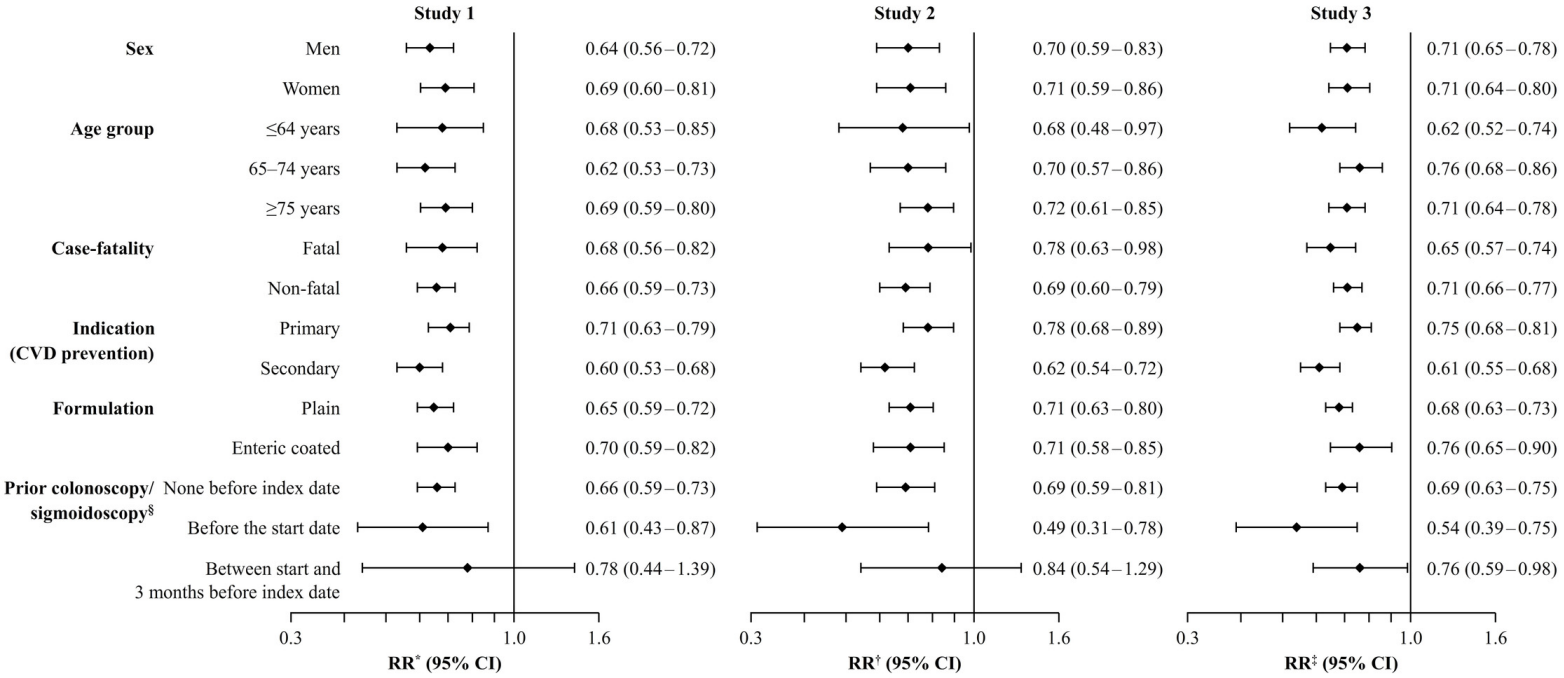
RRs (95% CIs) for risk of CRC with current use of low-dose aspirin (versus never use) by patient subgroup for each study design. In the adjusted models, smoking, BMI, use of insulins, NSAIDs, oral-steroids, paracetamol, and low-dose aspirin were ascertained any time before the index date. *Adjusted by matched factors, number of PCP visits in the year before the index date, smoking, insulin, NSAIDs, BMI, and oral steroids. ^†^Adjusted by matched factors, number of PCP visits in the year before the index date, smoking, BMI, NSAIDs, and paracetamol. ^‡^Adjusted by matched factors, number of PCP visits in the year before the index date, smoking, NSAIDs and BMI. ^§^Record of a GI adenoma, sigmoidoscopy or colonoscopy. BMI, body mass index; CI, confidence interval; CRC, colorectal cancer; CVD, cardiovascular disease; NSAIDs, non-steroidal antiinflammatory drugs; RR, rate ratio; PCP, primary care practitioner.

Current use of low-dose aspirin was associated with a statistically significant reduction in risk of CRC in each study design; RR 0.66 (95% CI: 0.60–0.73) in Study 1, 0.71 (95% CI: 0.63–0.80) in Study 2 and 0.69 (95% CI: 0.64–0.74) in Study 3 (Fig 3). Reduced risks of CRC were seen throughout treatment duration, and no dose–response relationships were observed. The RR of CRC with current use of low-dose aspirin is shown by patient subgroups in Fig 4. Using all three study designs, significantly reduced risks of CRC were seen when low-dose aspirin was used for primary or secondary cardiovascular disease prevention, for both fatal and non-fatal cases, for colon or rectal CRC, for both sexes, and across all age groups. Age did not modify the protective effect of low-dose aspirin (*p* for interaction = 0.27, 0.23 and 0.53 in the three studies, respectively), and no differences were observed between men and women (*p* for interaction = 0.24, 0.98 and 0.98, respectively). In addition, current use of low-dose aspirin was associated with a reduced risk of CRC among patients with or without previous colonoscopy/sigmoidoscopy procedures (Fig 4) and among patients with 10 or more PCP visits in the year before the index date: RR 0.74 (95% CI: 0.65–0.83) in Study 1, RR 0.80 (95% CI: 0.70–0.91) in Study 2 and RR 0.74 (95% CI: 0.68–0.80) in Study 3.

No association between current use of paracetamol monotherapy and CRC was observed in any of the three study designs, RRs: 1.04 (95% CI: 0.91–1.19) in Study 1, 1.00 (95% CI: 0.88–1.14) in Study 2 and 1.00 (95% CI: 0.93–1.09) in Study 3. Compared with non-use of DAT, current use of clopidogrel monotherapy was associated with a reduction in CRC risk, RRs were 0.78 (95% CI: 0.42–1.44), 0.56 (95% CI: 0.39–0.81) and 0.68 (95% CI: 0.53–0.86) in Study 1, 2 and 3. Corresponding estimates for current use of DAT were 0.88 (95% CI: 0.63–1.22), 0.65 (95% CI: 0.46–0.91) and 0.65 (95% CI: 0.48–0.88).

## Discussion

Using data from a validated UK primary care database, our results support a 30% protective effect of low-dose aspirin against the development of CRC. This effect was consistent across our three studies, each adopting a different study design, supporting previous findings. Our findings also suggest this protective effect is present across patient subgroups. In each study design, a daily dose of 75 mg/day was effective in reducing CRC risk in line with previous findings [5]. This is an important and potentially beneficial public health finding because aspirin doses of less than 100 mg are sufficient to reduce the risk of thrombotic cardiovascular events, while the risk of aspirin-associated gastrointestinal bleeding is dose-dependent and is lower with daily doses of 75 mg than with 300 mg [14–16]. We did not observe any dose–response relationships between low-dose aspirin and CRC; however, more than 90% of aspirin use in each study was at a dose of 75 mg/day, leaving insufficient power to assess the higher doses evaluated. While overall CRC incidence rates from Studies 1 and 2 were higher due to the age differences at start of follow up, CRC occurrence was similar across our three studies when reporting age- and sex-specific rates, with a pattern of increased incidence with age in both sexes and consistently higher rates in men. Incidence rates from Study 3, using a cohort of relatively ‘unselected’ patients are, however, the most representative estimates, and are in line with national statistics from the UK during the study period [17–19].

Owing to the very similar results across our three studies, despite the variation in the characteristics of the study cohort(s) between the three studies, we feel it is unlikely that the observed protective effect of low-dose aspirin is explained by bias. All three studies adopted new-user study designs [20] that would have helped to minimize the survival bias present among prevalent users. The design of Study 1 would have helped minimize potential bias arising from differences between users and non-users of low-dose aspirin, which may have been difficult to otherwise control, while use of a ‘neutral’ drug—paracetamol—in Study 2 minimized any healthy user bias. In fact, we found that in Study 2, the new-users of paracetamol at start of follow-up cohort was less healthy in terms of several comorbidities and lifestyle factors than the new users of low-dose aspirin at start of follow-up cohort. Moreover, the lack of association between paracetamol and CRC observed in the three studies further reinforces the validity of this drug for use as an active comparator group.

Our results have high external validity because data in THIN has been shown to be representative of the UK general population [8]. In addition, the source population for each study included a broad range of real-world patients, including those with gastrointestinal disorders. Previously, we have found the Dukes Stages of CRC cases in Study 1 to be broadly consistent with national data [11] supporting their representativeness to cases in the general population. We have also previously validated cases from Study 1 through linkage to hospitalization data and through PCP questionnaires for a smaller sample [11], finding high confirmation rates. Characteristics of cases and controls, in terms of healthcare use, comorbidities and lifestyle factors, were similar across the three studies, supporting the likelihood that cases in Studies 2 and 3 are also likely to have a high level of validity and be representative of those in the general population.

In the UK, low-dose aspirin can be obtained over-the-counter (OTC), yet misclassification of aspirin exposure due to unrecorded OTC use is likely to be minimal as shown previously through a survey of PCPs contributing data to THIN [21]. Any misclassification because of non-adherence would have been non-differential and biased the risk estimates towards the null. Paracetamol is also available as an OTC medication in the UK and similarly any misclassification is likely to be non-differential between cases and controls. Residual confounding, however, cannot be excluded despite being able to control for several confounders in our analyses. Some risk factors for CRC, such as a positive family history and red meat intake are not recorded in the database and so we could not include these variables in our analyses; however, they are unlikely to be related to aspirin exposure and confound the associations found.

In conclusion, we have found that new use of low-dose aspirin is associated with a significant 30% reduced risk of CRC, consistent across patient subgroups. The similarity of our results between studies adopting different study designs makes it unlikely that the findings can be explained by selection bias.

## Supporting Information

S1 Table Read codes for colorectal cancer. (DOC)

S2 Table Frequency of low-dose aspirin use in each study design. (DOC)

S1 Text Supplementary methods. (DOC)
