# Supplementary material for: The Protective Effect of Low-Dose Aspirin against Colorectal Cancer Is Unlikely Explained by Selection Bias: Results from Three Different Study Designs in Clinical Practice
Source: PLoS One. 2016 Jul 18;11(7):e0159179. doi: 10.1371/journal.pone.0159179 (PMC4948817; doi:10.1371/journal.pone.0159179)
Supplement: S1 Table — (DOC) [file pone.0159179.s001.doc]

**S1 Table. Read codes for CRC.**

| Dukes Stage A |
| --- |
| Stage a |
| Stage a |
| Stage b |
| Stage b |
| Stage b |
| Stage c |
| Stage c |
| Stage c |
| Stage d |
| Stage d |
| Stage d |
| Colon |
| Colon |
| Colon |
| Rectum |
| Rectum |
| Rectum |
| Fatal |
| Fatal |
| Fatal |
| Non-fatal |
| Non-fatal |
| Non-fatal |
| men |
| men |
| men |
| women |
| women |
| women |
| Age <=64 years |
| Age <=64 years |
| Age <=64 years |
| Age 65–74 years |
| Age 65–74 years |
| Age 65–74 years |
| Age >= 75 years |
| Age >= 75 years |
| Age >= 75 years |
| Primary CVD prevention |
| Primary CVD prevention |
| Primary CVD prevention |
| Secondary CVD prevention |
| Secondary CVD prevention |
| Secondary CVD prevention |
| Upper GI antecedents |
| Upper GI antecedents |
| Upper GI antecedents |
| No upper Gi antecedents |
| No upper Gi antecedents |
| No upper Gi antecedents |
| No prior bowel investigation * |
| No prior bowel investigation * |
| No prior bowel investigation * |
| Bowel investigation before start date |
| Bowel investigation before start date |
| Bowel investigation before start date |
| Bowel investigation between start date and 3 months before index date |
| Bowel investigation between start date and 3 months before index date |
| Bowel investigation between start date and 3 months before index date |

| Dukes Stage B |
| --- |
| Stage a |
| Stage a |
| Stage b |
| Stage b |
| Stage b |
| Stage c |
| Stage c |
| Stage c |
| Stage d |
| Stage d |
| Stage d |
| Colon |
| Colon |
| Colon |
| Rectum |
| Rectum |
| Rectum |
| Fatal |
| Fatal |
| Fatal |
| Non-fatal |
| Non-fatal |
| Non-fatal |
| men |
| men |
| men |
| women |
| women |
| women |
| Age <=64 years |
| Age <=64 years |
| Age <=64 years |
| Age 65–74 years |
| Age 65–74 years |
| Age 65–74 years |
| Age >= 75 years |
| Age >= 75 years |
| Age >= 75 years |
| Primary CVD prevention |
| Primary CVD prevention |
| Primary CVD prevention |
| Secondary CVD prevention |
| Secondary CVD prevention |
| Secondary CVD prevention |
| Upper GI antecedents |
| Upper GI antecedents |
| Upper GI antecedents |
| No upper Gi antecedents |
| No upper Gi antecedents |
| No upper Gi antecedents |
| No prior bowel investigation * |
| No prior bowel investigation * |
| No prior bowel investigation * |
| Bowel investigation before start date |
| Bowel investigation before start date |
| Bowel investigation before start date |
| Bowel investigation between start date and 3 months before index date |
| Bowel investigation between start date and 3 months before index date |
| Bowel investigation between start date and 3 months before index date |

| Dukes Stage C |
| --- |
| Stage a |
| Stage a |
| Stage b |
| Stage b |
| Stage b |
| Stage c |
| Stage c |
| Stage c |
| Stage d |
| Stage d |
| Stage d |
| Colon |
| Colon |
| Colon |
| Rectum |
| Rectum |
| Rectum |
| Fatal |
| Fatal |
| Fatal |
| Non-fatal |
| Non-fatal |
| Non-fatal |
| men |
| men |
| men |
| women |
| women |
| women |
| Age <=64 years |
| Age <=64 years |
| Age <=64 years |
| Age 65–74 years |
| Age 65–74 years |
| Age 65–74 years |
| Age >= 75 years |
| Age >= 75 years |
| Age >= 75 years |
| Primary CVD prevention |
| Primary CVD prevention |
| Primary CVD prevention |
| Secondary CVD prevention |
| Secondary CVD prevention |
| Secondary CVD prevention |
| Upper GI antecedents |
| Upper GI antecedents |
| Upper GI antecedents |
| No upper Gi antecedents |
| No upper Gi antecedents |
| No upper Gi antecedents |
| No prior bowel investigation * |
| No prior bowel investigation * |
| No prior bowel investigation * |
| Bowel investigation before start date |
| Bowel investigation before start date |
| Bowel investigation before start date |
| Bowel investigation between start date and 3 months before index date |
| Bowel investigation between start date and 3 months before index date |
| Bowel investigation between start date and 3 months before index date |

| Dukes Stage D |
| --- |
| Stage a |
| Stage a |
| Stage b |
| Stage b |
| Stage b |
| Stage c |
| Stage c |
| Stage c |
| Stage d |
| Stage d |
| Stage d |
| Colon |
| Colon |
| Colon |
| Rectum |
| Rectum |
| Rectum |
| Fatal |
| Fatal |
| Fatal |
| Non-fatal |
| Non-fatal |
| Non-fatal |
| men |
| men |
| men |
| women |
| women |
| women |
| Age <=64 years |
| Age <=64 years |
| Age <=64 years |
| Age 65–74 years |
| Age 65–74 years |
| Age 65–74 years |
| Age >= 75 years |
| Age >= 75 years |
| Age >= 75 years |
| Primary CVD prevention |
| Primary CVD prevention |
| Primary CVD prevention |
| Secondary CVD prevention |
| Secondary CVD prevention |
| Secondary CVD prevention |
| Upper GI antecedents |
| Upper GI antecedents |
| Upper GI antecedents |
| No upper Gi antecedents |
| No upper Gi antecedents |
| No upper Gi antecedents |
| No prior bowel investigation * |
| No prior bowel investigation * |
| No prior bowel investigation * |
| Bowel investigation before start date |
| Bowel investigation before start date |
| Bowel investigation before start date |
| Bowel investigation between start date and 3 months before index date |
| Bowel investigation between start date and 3 months before index date |
| Bowel investigation between start date and 3 months before index date |

| Colon |
| --- |
| Stage a |
| Stage a |
| Stage b |
| Stage b |
| Stage b |
| Stage c |
| Stage c |
| Stage c |
| Stage d |
| Stage d |
| Stage d |
| Colon |
| Colon |
| Colon |
| Rectum |
| Rectum |
| Rectum |
| Fatal |
| Fatal |
| Fatal |
| Non-fatal |
| Non-fatal |
| Non-fatal |
| men |
| men |
| men |
| women |
| women |
| women |
| Age <=64 years |
| Age <=64 years |
| Age <=64 years |
| Age 65–74 years |
| Age 65–74 years |
| Age 65–74 years |
| Age >= 75 years |
| Age >= 75 years |
| Age >= 75 years |
| Primary CVD prevention |
| Primary CVD prevention |
| Primary CVD prevention |
| Secondary CVD prevention |
| Secondary CVD prevention |
| Secondary CVD prevention |
| Upper GI antecedents |
| Upper GI antecedents |
| Upper GI antecedents |
| No upper Gi antecedents |
| No upper Gi antecedents |
| No upper Gi antecedents |
| No prior bowel investigation * |
| No prior bowel investigation * |
| No prior bowel investigation * |
| Bowel investigation before start date |
| Bowel investigation before start date |
| Bowel investigation before start date |
| Bowel investigation between start date and 3 months before index date |
| Bowel investigation between start date and 3 months before index date |
| Bowel investigation between start date and 3 months before index date |

| Rectum |
| --- |
| Stage a |
| Stage a |
| Stage b |
| Stage b |
| Stage b |
| Stage c |
| Stage c |
| Stage c |
| Stage d |
| Stage d |
| Stage d |
| Colon |
| Colon |
| Colon |
| Rectum |
| Rectum |
| Rectum |
| Fatal |
| Fatal |
| Fatal |
| Non-fatal |
| Non-fatal |
| Non-fatal |
| men |
| men |
| men |
| women |
| women |
| women |
| Age <=64 years |
| Age <=64 years |
| Age <=64 years |
| Age 65–74 years |
| Age 65–74 years |
| Age 65–74 years |
| Age >= 75 years |
| Age >= 75 years |
| Age >= 75 years |
| Primary CVD prevention |
| Primary CVD prevention |
| Primary CVD prevention |
| Secondary CVD prevention |
| Secondary CVD prevention |
| Secondary CVD prevention |
| Upper GI antecedents |
| Upper GI antecedents |
| Upper GI antecedents |
| No upper Gi antecedents |
| No upper Gi antecedents |
| No upper Gi antecedents |
| No prior bowel investigation * |
| No prior bowel investigation * |
| No prior bowel investigation * |
| Bowel investigation before start date |
| Bowel investigation before start date |
| Bowel investigation before start date |
| Bowel investigation between start date and 3 months before index date |
| Bowel investigation between start date and 3 months before index date |
| Bowel investigation between start date and 3 months before index date |

| Fatal |
| --- |
| Stage a |
| Stage a |
| Stage b |
| Stage b |
| Stage b |
| Stage c |
| Stage c |
| Stage c |
| Stage d |
| Stage d |
| Stage d |
| Colon |
| Colon |
| Colon |
| Rectum |
| Rectum |
| Rectum |
| Fatal |
| Fatal |
| Fatal |
| Non-fatal |
| Non-fatal |
| Non-fatal |
| men |
| men |
| men |
| women |
| women |
| women |
| Age <=64 years |
| Age <=64 years |
| Age <=64 years |
| Age 65–74 years |
| Age 65–74 years |
| Age 65–74 years |
| Age >= 75 years |
| Age >= 75 years |
| Age >= 75 years |
| Primary CVD prevention |
| Primary CVD prevention |
| Primary CVD prevention |
| Secondary CVD prevention |
| Secondary CVD prevention |
| Secondary CVD prevention |
| Upper GI antecedents |
| Upper GI antecedents |
| Upper GI antecedents |
| No upper Gi antecedents |
| No upper Gi antecedents |
| No upper Gi antecedents |
| No prior bowel investigation * |
| No prior bowel investigation * |
| No prior bowel investigation * |
| Bowel investigation before start date |
| Bowel investigation before start date |
| Bowel investigation before start date |
| Bowel investigation between start date and 3 months before index date |
| Bowel investigation between start date and 3 months before index date |
| Bowel investigation between start date and 3 months before index date |

|  |
| --- |
|  |
|  |
|  |
|  |
|  |
|  |
|  |
|  |
|  |
|  |
|  |
|  |
|  |
|  |
|  |
|  |
|  |
|  |
|  |
|  |
|  |
|  |
|  |
|  |
|  |
|  |
|  |
|  |
|  |
|  |
|  |
|  |
|  |
|  |
|  |
|  |
|  |
|  |
|  |
|  |
|  |
|  |
|  |
|  |
|  |
|  |
|  |
|  |
|  |
|  |
|  |
|  |
|  |
|  |
|  |
|  |
|  |
|  |
|  |

| **Read** | **Descriptor** |
| --- | --- |
| B13..00 | Malignant neoplasm of colon |
| B130.00 | Malignant neoplasm of hepatic flexure of colon |
| B131.00 | Malignant neoplasm of transverse colon |
| B132.00 | Malignant neoplasm of descending colon |
| B133.00 | Malignant neoplasm of sigmoid colon |
| B134.00 | Malignant neoplasm of caecum |
| B134.11 | Carcinoma of caecum |
| B136.00 | Malignant neoplasm of ascending colon |
| B137.00 | Malignant neoplasm of splenic flexure of colon |
| B138.00 | Malignant neoplasm, overlapping lesion of colon |
| B13y.00 | Malignant neoplasm of other specified sites of colon |
| B13z.00 | Malignant neoplasm of colon NOS |
| B13z.11 | Colonic cancer |
| B14..00 | Malignant neoplasm of rectum, rectosigmoid junction and anus |
| B140.00 | Malignant neoplasm of rectosigmoid junction |
| B141.00 | Malignant neoplasm of rectum |
| B141.11 | Carcinoma of rectum |
| B141.12 | Rectal carcinoma |
| B14y.00 | Malig neop other site rectum, rectosigmoid junction and anus |
| B14z.00 | Malignant neoplasm rectum, rectosigmoid junction and anus NOS |
| ZV10017 | [V]Personal history of malignant neoplasm of rectum |

Note: In Study 1, two additional Read codes (8Hn4.00 ‘fast track referral suggestive of a possible CRC malignancy’ and 9Np7.00 ‘seen in fast track suspected colorectal cancer clinic’) were used to identify potential cases of CRC through computerized searches. However, following manual review of the records (including the free-text comments) of patients identified with these two codes, none were subsequently deemed to have CRC. In addition, after removing these patients from the CRC case set in Study 1, 86.4% of all other patients identified with a Read code for CRC were considered to be a case following the manual review process, and thus these two codes were not subsequently included in the CRC Read code list in Studies 2 and 3.
