# Supplementary material for: The Protective Effect of Low-Dose Aspirin against Colorectal Cancer Is Unlikely Explained by Selection Bias: Results from Three Different Study Designs in Clinical Practice
Source: PLoS One. 2016 Jul 18;11(7):e0159179. doi: 10.1371/journal.pone.0159179 (PMC4948817; doi:10.1371/journal.pone.0159179)
Supplement: S2 Table — (DOC) [file pone.0159179.s002.doc]

**S2 Table. Frequency of low-dose aspirin use in each study design.**

|  | **Study 1** | | | **Study 2** | | | | | **Study 3** | | | |
| --- | --- | --- | --- | --- | --- | --- | --- | --- | --- | --- | --- | --- |
| **Low-dose aspirin use** | **Cases**  **N=3033**  **n (%)** | | **Controls**  **N=10,000**  **n (%)** | | **Cases**  **N=3174**  **n (%)** | | **Controls**  **N=10,000**  **n (%)** | | | **Cases**  **N=12,333**  **n (%)** | | **Controls**  **N=20,000**  **n (%)** |
| **Recency** |  | |  | |  | |  | | |  | |  |
| Non-use | 1247 (41.1) | | 3557 (35.6) | | 1152 (36.3) | | 3597 (36.0) | | | 9818 (79.6) | | 15,987 (79.9) |
| Current use | 1255 (41.4) | | 4562 (45.6) | | 1305 (41.1) | | 4462 (44.6) | | | 1658 (13.4) | | 2782 (13.9) |
| Recent/past use | 531 (17.5) | | 1881 (18.8) | | 717 (22.6) | | 1941 (19.4) | | | 857 (6.9) | | 1231 (6.2) |
| **Among current users** |  |  | | | |  | |  | | |  | |
| **Daily dose** |  | |  | |  | |  | | |  | |  |
| 75 mg | 1137 (37.5) | | 4128 (41.3) | | 1202 (37.9) | | 4105 (41.1) | | | 1514 (12.3) | | 2513 (12.6) |
| 150 mg | 107 (3.5) | | 402 (4.0) | | 91 (2.9) | | 310 (3.1) | | | 127 (1.0) | | 235 (1.2) |
| 300 mg | 11 (0.4) | | 32 (0.3) | | 12 (0.4) | | 47 (0.5) | | | 17 (0.1) | | 34 (0.2) |
| **Formulation** |  | |  | |  | |  | | |  | |  |
| Plain | 1,008 (33.2) | | 3,716 (37.2) | | 1099 (34.6) | | 3763 (37.6) | | | 1411 (11.4) | | 2404 (12.0) |
| Enteric coated | 247 (8.1) | | 846 (8.5) | | 206 (6.5) | | 699 (7.0) | | | 247 (2.0) | | 378 (1.9) |
| **Duration of use** |  | |  | |  | |  | | |  | |  |
| <1 year | 433 (14.3) | | 1,430 (14.3) | | 324 (10.2) | | 986 (9.9) | | | 472 (3.8) | | 701 (3.5) |
| 1–5 years | 632 (20.8) | | 2,370 (23.7) | | 722 (22.7) | | 2517 (25.2) | | | 890 (7.2) | | 1581 (7.9) |
| **≥**5 years | 190 (6.3) | | 762 (7.6) | | 259 (8.2) | | 959 (9.6) | | | 296 (2.4) | | 500 (2.5) |
| **Indication  (CVD prevention)** |  | |  | |  | |  | | |  | |  |
| Primary | 746 (24.6) | | 2560 (25.6) | | 793 (25.0) | | 2527 (25.3) | | | 1014 (8.2) | | 1604 (8.0) |
| Secondary | 509 (16.8) | | 2002 (20.0) | | 512 (16.1) | | 1935 (19.4) | | | 644 (5.2) | | 1178 (5.9) |
| **Colonoscopy/sigmoidoscopy^*^** |  | |  | |  | |  | | |  | |  |
| None ever before index date | 1101 (36.3) | | 3980 (39.8) | | 741 (23.3) | | 3855 (38.6) | | | 983 (8.0) | | 2422 (12.1) |
| Before start date | 81 (2.7) | | 385 (3.9) | | 83 (2.6) | | 380 (3.8) | | | 63 (0.5) | | 170 (0.9) |
| Between start date and 3 months prior index date | 58 (1.9) | | 179 (1.8) | | 103 (3.2) | | 215 (2.2) | | | 170 (1.4) | | 205 (1.0) |
| **Fatal/non-fatal case** |  | |  | |  | |  | | |  | |  |
| Fatal | 255 (8.4) | | N/A | | 269 (8.5) | | N/A | | | 383 (3.1) | | N/A |
| Non-fatal | 1000 (33.0) | | N/A | | 1036 (32.6) | | N/A | | | 1275 (10.4) | | N/A |
| **Sex** |  | |  | |  | |  | | |  | |  |
| Male | 779 (25.7) | | 2854 (28.5) | | 860 (27.1) | | 2902 (29.0) | | | 1046 (8.5) | | 1735 (8.7) |
| Female | 476 (15.7) | | 1708 (17.1) | | 445 (14.0) | | 1560 (15.6) | | | 612 (5.0) | | 1047 (5.3) |
| **Age group (years)** |  | |  | |  | |  | | |  | |  |
| ≤64 | 210 (6.9) | | 747 (7.5) | | 215 (6.8) | | 698 (7.0) | | | 254 (2.1) | | 381 (1.9) |
| 65–74 | 482 (15.9) | | 1807 (18.1) | | 518 (16.3) | | 1731 (17.3) | | | 630 (5.1) | | 1015 (5.1) |
| ≥75 | 563 (18.6) | | 2008 (20.1) | | 572 (18.0) | | 2033 (20.3) | | | 774 (6.3) | | 1386 (6.9) |

**Numbers and percentages are among current low-dose aspirin users unless otherwise stated.**

**^*^**Record of a GI adenoma, colonoscopy or sigmoidoscopy.

CVD, cardiovascular disease.
