# Supplementary material for: The Protective Effect of Low-Dose Aspirin against Colorectal Cancer Is Unlikely Explained by Selection Bias: Results from Three Different Study Designs in Clinical Practice
Source: PLoS One. 2016 Jul 18;11(7):e0159179. doi: 10.1371/journal.pone.0159179 (PMC4948817; doi:10.1371/journal.pone.0159179)
Supplement: S1 Text — (DOC) [file pone.0159179.s003.doc]

# S1 Text. Supplementary methods

## Eligibility criteria to enter the source population

Before being able to enter the source population for each study, individuals were required to meet the following eligibility criteria:

- enrollment with their primary care practitioner (PCP) for at least 2 years

- a computerized prescription history dating back at least 1 year

- at least one healthcare contact within the last 3 years.

These criteria served to ensure that members of the study population were making use of healthcare services within their practice and had some extent of historical information. The date that an individual met all eligibility criteria was considered as the study entry date.

Individuals were excluded if they were aged 70 years or more, with a follow-up longer than 1 year and fewer than two recorded consultations with a PCP during their entire follow-up (proxy for incomplete and invalid data recording).

## Further details on the identification of risk factors

In addition to low-dose aspirin, medications evaluated included clopidogrel, dual antiplatelet therapy with low-dose aspirin/clopidogrel, antihypertensives, statins, antidiabetics, non-steroidal anti-inflammatory drugs, oral steroids, warfarin, thienopyridines and proton pump inhibitors. Duration of therapy was calculated by summing the individual duration of all consecutive prescriptions with gaps in treatment of more than 90 days considered genuine breaks in treatment. In addition, for low-dose aspirin, cumulative duration was calculated by summing the days of low-dose aspirin supply irrespective of gaps between prescriptions. We ascertained the average daily dose of low-dose aspirin by using the information on the dose prescribed together with the instructions. To identify the indication for low-dose aspirin, we used a computer algorithm that searched THIN for Read codes suggestive of cardiovascular disease (CVD; comprising myocardial infarction, unstable angina, peripheral artery disease and ischaemic heart disease) from any time before the first low-dose aspirin prescription and up to 30 days after. Patients with a relevant Read code within this time frame were classed as having received low-dose aspirin for secondary CVD prevention and all other patients were assumed to have received low-dose aspirin for primary CVD prevention. Information was also extracted from the database regarding previous bowel investigations (a record of adenoma, colonoscopy or sigmoidoscopy) or upper gastrointestinal disorders (dyspepsia, or complicated or uncomplicated peptic ulcer) any time before the start date.

## Statistical analysis

Incidence rates of CRC with 95% confidence intervals were calculated in each study expressed as number of CRC cases per 10,000 person-years. Stratified analyses were performed according to low-dose aspirin dose (75 mg, 150 mg or 300 mg), duration of use, indication and formulation (plain or enteric coated), case-fatality status (deaths within the first year after CRC diagnosis were considered to be fatal cases), age, gender and previous bowel investigation.
